# A new guide for basic psychological support for persons affected by neglected tropical diseases: A peer support tool suitable for persons with a diagnosis of leprosy and lymphatic filariasis

**DOI:** 10.1371/journal.pntd.0011945

**Published:** 2025-01-09

**Authors:** Pradeepta K. Nayak, Charles D. Mackenzie, Ashok Agarwal, Robin van Wijk, Marente M. Mol, Julian Eaton, Maya Semrau, Carmen Valle-Trabadelo, Gauri S. Kaloiya, Alok Pratap, Jayashree P. Kunju, Suma Krishnasastry, Astri Ferdiana, Ariana Marastuti, Rohit K. Tiwari, Wim H. van Brakel

**Affiliations:** 1 NLR India, New Delhi, India; 2 NTD Support Center, Task Force for Global Health, Decatur, Georgia, United States of America; 3 RLMF, The End Fund, New York, New York, United States of America; 4 NLR | until No Leprosy Remains, Amsterdam, The Netherlands; 5 Erasmus University Medical Center, Department: Public Health, Rotterdam, The Netherlands; 6 Centre for Global Mental Health, London School of Hygiene and Tropical Medicine LSHTM, London, United Kingdom; 7 CBM Global, Van Heuven Goedhartlaan, Amstelveen, Netherlands; 8 Centre for Global Health Research, Brighton and Sussex Medical School, Falmer, Brighton, United Kingdom; 9 IASC MHPSS Reference Group, IFRC Reference Centre for Psychosocial Support, Copenhagen, Denmark; 10 National Drug Dependence Treatment Centre (NDDTC), All India Institute of Medical Sciences (AIIMS), Ansari Nagar, New Delhi, India; 11 Department of Psychiatry, Central Institute of Psychiatry, Ranchi, India; 12 Global Leprosy Alliance for Zero Stigma (GLAZS), Bangalore, India; 13 Filariasis research Centre, WHO Collaborating Centre for LF MMDP, Government T D Medical College, Alappuzha, Kerala, India; 14 Department of Public Health, Faculty of Medicine Universitas Mataram, Mataram, Indonesia; 15 NLR Indonesia, Jakarta, Indonesia; 16 Department of Psychology, Gadjah Mada University, Jalan Sosio Humaniora Bulaksumur, Yogyakarta, Indonesia; University of California Berkeley, UNITED STATES OF AMERICA

## Abstract

**Background:**

People with disabilities due to neglected tropical diseases (NTDs), such as leprosy and lymphatic filariasis (LF), often encounter situations of stigma and discrimination that significantly impact their mental wellbeing. Mental wellbeing services are often not available at the peripheral level in NTD-endemic countries, and there is a need for such services. Basic psychological support for persons with NTDs (BPS-N) from peers is an important potential solution for addressing mental wellbeing problems. As there was no written document advising delivery of such support, NLR India brought experts together to develop a new guide. This paper describes the process used in developing the guide and provides information about its content.

**Methods:**

As a qualitative and participatory methodology, more than 10 meetings and workshops were held to consider the suitability of existing guides for chronic stress in NTDs and develop a new guide through consensus and adaptations; attendees included both technical experts and affected persons. The first meeting was a 3-day virtual workshop held on 9–11 June 2020, followed by other online meetings. The BPS-N guide development happened during the COVID-19 lockdowns. The Psychological First Aid (PFA) package of WHO was selected as a suitable basic model for adaptation. Aspects of the Rights-Based Counselling intervention were also integrated into the new guide. Two teams were formed for drafting and reviewing the guide.

**Results:**

All suggested changes were discussed, and a consensus reached for developing the document. The affected persons contextualized the content for ensuring its relevance and practicality. The new BPS-N guide was simple, professionally sound, ethical, adequate, and appropriate. The guide promotes knowledge, skills, compassion, and action among peer supporters.

**Conclusion:**

The new guide, through regular trainings, behavior change, and action principles will likely provide much-needed services. It is important that the new guide be now tested, and modifications made if needed.

## Author summary

Mental health services are often not available at the community levels in NTD-endemic countries; this is a vital felt need. While basic psychological support from peers could be an important solution, NLR India took efforts and developed a new guide that has shown effectiveness. We aimed to describe the process of development of the guide.

A qualitative approach was taken in weighing the values of various guides and arriving at a consensus, during meetings sponsored by NLR India, for developing the new guide through adaptations. The Psychological First Aid guide of WHO was selected as a suitable basic model. Aspects of the Rights-Based Counselling intervention procedures were also used for adaptation.

A review team verified the final document—Basic Psychological Support for Neglected Tropical Diseases (BPS-N)—to ensure it was simple, adequate, and appropriate; affected persons contributed to its practicality.

The guide promotes awareness, empathy, ethics, willingness and abilities among the peer supporters, community and health staff. Its use would reduce stigma, improve confidence, compassion, communication, participation and mental wellbeing of those with NTDs. Regular training, and actions advised by this new guide may change the lives of the affected millions.

## Introduction

Neglected tropical diseases (NTDs) are a group of twenty, predominantly communicable, chronic, and disabling conditions [1]. Certain NTDs such as leprosy and lymphatic filariasis (LF) may cause, in addition to extensive physical disabilities, both psychosocial, and mental health impairment if not treated in time [2]. Persons affected by NTDs are prone to social stigmatization and discrimination, due to the physical impairments and disfigurements that accompany some NTDs [3]. The stigma associated with NTDs causes an enormous social and psychological burden in terms of social exclusion, reduced quality of life, and poor mental health–particularly where skin is affected [4]. A study in India found that around 50% of people affected with leprosy or LF reported experiences of depression and anxiety, with disability found to be the main contributing factor for mental wellbeing issues, with exclusion, income, gender and age having a lower effect [5]. These observations were supported by a second study in India that found a high prevalence of depression and anxiety among the persons affected by leprosy [6]. The prevalence of mental health issues amongst people affected by NTDs is far higher than that of the general population, and stigma and social exclusion seem to be the major mechanisms through which this manifests [7].

The Psychological First Aid (PFA) intervention, developed by the World Health Organisation [8,9] has been promoted as a suitable tool for enhancing mental wellbeing of persons facing acute trauma or humanitarian crises, but there is insufficient evidence for its effectiveness in providing basic psychological support in broader contexts such as in NTDs, though there is reason to believe such skills among frontline workers or peers would be valuable [10]. On the other hand, engaging in peer support interventions has been found to produce modest, though consistent, results suggesting that these activities may also be effective for mental health [11]. Indeed, a trial in Indonesia assessing the impact of a peer counselling intervention to reduce leprosy-related stigma showed this to be effective [12], and the authors recommended for further validation of this approach through application on a larger scale, and modifications to make it more sustainable.

An exploratory study was carried out between January 2020 and May 2022 by NLR India that involved adaptation of the PFA for use with persons with disabilities due to NTDs, especially leprosy and LF. This study underscored the importance of mental health issues, stigma, disability, discrimination, and exclusion in NTDs, and of the need to develop a psychological support approach suitable for use by peers of the persons affected by leprosy and LF. A before-and-after intervention study was conducted to provide proof-of-concept that basic psychological support tool for people affected by neglected tropical diseases (BPS-N) can be used by peer supporters (PSs) to reduce stigma, improve mental wellbeing and participation among their clients. The study showed that the BPS-N intervention had a positive impact in the short-term. There were 75 persons with disabilities (32 due to leprosy and 43 due to LF) enrolled in the intervention and supported by 13 peer supporters (6 leprosy affected and 7 LF affected). Each peer supporter was responsible for 5–6 clients providing BPS-N. After a period of three months, there were significant improvements in mental wellbeing, and significant reductions in mean stigma and depression levels [13]. As a follow-up to the above proof-of-concept study, another study explored the impact of the new peer support-based BPS-N intervention in India, with the striking finding that there was an increased positive effect on stigma in a five-month follow-up compared with the immediate post-intervention scores. With this follow-up study, further evidence of the BPS-N based peer support intervention has been added [14]. These early studies indicate that this peer driven protocol, the BPS-N guide, is a useful addition to the care for those affected by stigmatizing conditions, and specifically leprosy and lymphatic filariasis.

The pre-intervention activity of the proof-of-concept study consisted of the development of the BPS-N guide [13]. The need for development of this guide was felt because there was no written protocol on this. This paper intends to report the steps taken to create a consensus-based appropriate tool for peer-delivered psychological support, the BPS-N guide, and its content, that can be used to make positive changes in the minds and lives of millions of people affected by NTDs.

## Methods

### Ethics statement

Ethical approval was obtained from the Ethical Committee, Institute of Medical Sciences, Banaras Hindu University (Dean/2020/EC/882 dated 21.01.2020). The formal written consents were obtained from the participants of the BPS-N intervention study. However, for development of the BPS-N guide, which this paper is all about; the experts had expressed their verbal consent, during the interactions, workshop and meetings, to support the effort, and contribute their experience, insights and inputs.

### Goal

While NLR India had designed a mental wellbeing intervention to provide a community-based basic psychological support to the NTD affected persons through their peers, there was no written document on how to deliver this service. NLR India intended to develop a guide that advises on how to provide care, support treatment, reduce stigma, and improve mental wellbeing, social as well as work participation of persons affected by NTDs, and can prevent future mental health issues. It should address psycho-social problems and encourage a person-centred peer support system as community-based solution. This guide should encourage delivery of basic psychological support through quality, compassion, consistency, and timeliness, and build the capabilities of the peer supporters to serve their peer clients. NLR India was convinced that a new guide could be better developed and adapted through engagement of the relevant experts and affected persons for a consensus and contributions, and the PFA can be a suitable basic model.

### Strategies

NLR India adopted a qualitative and participatory methodology, using the steps—of consultation, consensus, collaboration, collection of qualitative information, compilation and adaptation, designing and printing—for developing the new guide on BPS-N. The main strategy was to engage experts in the areas of NTDs and mental health, and affected persons, through meetings and workshops, to discuss the reasons for developing the new guide, and obtain opinions and group consensus on its content. This also involved discussing the suitability of existing guides for chronic stress and mental health in persons affected by NTDs. The discussions were informed by the literature including the recent studies (as described in the introduction above), and by relevant documents such as those related to the WHO PFA [8,9,10]. An important sub-objective was to adapt the PFA for use for the persons affected by NTDs suffering from stigma and mental wellbeing issues. It was discussed that PFA was the only standard guide available for providing basic psychological support to persons in distress; however, the PFA was applicable for acute situations like war or earthquake and later adapted for Ebola, an acute infectious condition. Central to the discussions were also the key elements from the Rights-Based Counselling Module (RBCM), used in the peer counselling intervention in Indonesia [12], which could be included in the BPS-N document. Milestones were set for the deliberations by the meeting leaders that included discussion for the drafting, designing, testing and dissemination of the final product.

### Participants

The processes for development of the BPS-N guide happened during the COVID times. The lockdowns limited full engagement and left NLR India with the only option of online events. Apart from a three-day workshop, more than 10 virtual meetings were conducted for development of the guide, discussions and review; these meetings were of durations of 1–2 hours. The three-day virtual workshop was conducted to kick start the development of the BPS-N tool on 9–11 June 2020. The participants were identified and invited based on pre-selected criteria: (a) known experts on mental well-being; (b) previously engaged in development of the WHO PFA; (c) knowledge and working experience of leprosy and LF; (d) experts from India and outside; and (e) medical, psychological and programmatic experts. Each participant fulfilled at least two of the criteria. There were 27 international and national experts on NTDs together with mental wellbeing specialists who participated in the workshop. The group comprised of people representing international NGOs, hospitals, academia, government, and persons affected by NTDs. All the participants had agreed to contribute to the development of the guide by sharing their opinions, experience and expertise.

The participants identified the reasons affecting mental well-being of the persons affected by NTDs, primarily leprosy and LF in the context of India. The reasons were classified in different categories namely health conditions including disability, stigma, discrimination, deprivation and poverty, isolation, access inequality, social exclusion, and other socio-cultural and economic issues. It was followed by identifying interventions to address the problems. Undergoing the process, the group realized the need to develop new guidelines which address the identified needs through an easy-to-do method similar to the Look, Listen and Link approach of PFA. There was a consensus on the kind of information that needed to be collected and complied. The study investigators in discussion with the lead authors of PFA decided to adapt it for providing basic psychological support for leprosy and LF and the associated chronic situations. The investigators also decided to add important aspects from the Rights-Based Counselling Module (RBCM) used for persons affected by leprosy in Indonesia [12]; this led to condition-specific knowledge and information on human rights to be included in the guide. It was agreed that the resulting guide—by following training, appropriate behaviour change, and any actions based on the “Look-Listen-Link” principles—will likely provide much-needed mental wellbeing support.

The workshop participants were divided into two teams: a guidelines development team with 22 members and a review team of five members. The members of these teams of experts came from a variety of geographical regions and professional disciplines like public health (including NTDs), academia, global mental health, psychology, and psychiatry. The inclusion of the latter group was important to ensure appropriate contextualization of the content of the guide as was the inclusion of people affected by NTDs. Online participation, and face to face meetings (of NLR India staff) discussed the planned topics, using the documents and document links provided to all participants. The experts discussed, advised and agreed to include the topics like specific physical conditions; how to help the self and others; respecting rights, dignity, security; stress, readiness, rest and reflection. They recommended to use simple words, small sentences, define the difficult terms, and put the guidelines in a sequence, trying to put the first support first, and the next steps in a logical order. While it was discussed and decided to interweave the content thematically, milestones were set for the drafting, design, testing and dissemination of the final product.

#### Document management

Following the recommendations of the consensus meeting, the development team obtained permission from the Translation & Licensing Department of the World Health Organization to adapt and translate the PFA and publish it under a new name, with credit independent of WHO. Adaptations were made to change the focus from helping people in acute, sometimes life-threatening situations, to an instrument more appropriate for supporting people suffering from the chronic stresses typically associated with disability, stigma and poverty experienced by those affected by NTDs. The WHO PFA guide was helpful in laying out the chapters particularly with the users in mind i.e. the peer supporters, and other lay persons and frontline workers. Before being arranged in the guide, the contents were interwoven thematically—based on the issues, solutions, suggestions and interventions identified—with care taken to order the tips stepwise. As per the recommendation of the consensus meeting, disease-specific information on leprosy and LF were included in the tool, as was information on human rights particularly relevant to the situation of persons affected by NTDs. The guide development team members were central to the writing of the consensus guide using the previous iterations of the BSP-N document drafts, which were circulated regularly to the review team. The review team verified that the content was simple, correct, complete and fit for purpose and use by peer supporters. The feedback from the review team and the persons affected by NTDs was incorporated into the draft document after reaching consensus in the team. Consensus was achieved by mutual discussion and areas of concern settled through meaningful dialogue and deliberation. While the first complete draft was done by 18 June 2021, the second draft was shared by 12 July 2021. The content was finalized by 17 Aug 2021. The final consensus document was given to a graphic design firm to create culturally relevant and disability-inclusive artwork. The designing of the guide was finalised by NLR India in consultation with NLR International. The entire adaptation process and guide development work was coordinated by Dr. Ashok Agarwal, NLR India with support from Dr. Wim H. van Brakel of NLR International.

## Results

The workshop resulted in development of the BPS-N framework and contents of the new guide by obtaining group consensus. The BPS-N framework laid down the different chapters and their order. The consensus discussions, and subsequent reviews of the overall opinions, resulted in a final guide that fulfilled the defined goals. As proposed earlier, it was named the Basic Psychological Support for Neglected Tropical Diseases (BPS-N). In addition to the guide, a summary pocket guide and video were developed for training of the peer supporters. The English language guidebook and pocket guide were both translated into Hindi.

The guide and pocket guide are available at: https://www.infontd.org/practical-material/guide-basic-psychological-support-persons-affected-neglected-tropical-diseases

The InfoNTD video, Basic Psychological Support for Persons Affected by Neglected Tropical Diseases (BPS -N), is available on YouTube.com.

### BPS-N structure and content

The final BPS-N guide consists of an introductory chapter and four content chapters (Table 1). The introductory chapter defines mental health and gives basic information about NTDs in general, with more specific details about leprosy and LF. The chapter mentions how the two NTDs spread, their diagnosis, treatment and complications; it also mentions self-care, its importance and how to do self-care. It also provides guidance on COVID-19 and on how to take precautions and care of persons with NTDs when affected by COVID-19.

**Table 1:** Chapters of the BPS-N Guide.

**INTRODUCTION**	**UNDERSTANDING NEGLECTED TROPICAL DISEASES**
**CHAPTER 1**	**UNDERSTANDING BASIC PSYCHOLOGICAL SUPPORT**
**CHAPTER 2**	**HOW TO HELP RESPONSIBLY**
**CHAPTER 3**	**PROVIDING BASIC PSYCHOLOGICAL SUPPORT FOR NTDs**
**CHAPTER 4**	**CARING FOR YOURSELF AND YOUR COLLEAGUES**
**APPENDIX**	**POCKET GUIDE**

Chapter 1 describes how NTDs and their consequences affect people. The chapter also provides guidance on helping vulnerable people and defines what basic psychological support is. It explains when and where it is appropriate to provide BPS-N. As peer supporters are expected and urged to provide BPS-N, the chapter defines who a peer supporter is for the persons affected by NTDs. As safety, dignity and rights of the affected persons are extremely important, Chapter 2 explains these desired values, and provides tips to the peer supporters on how to ensure these with “do’s and don’ts”. This chapter also emphasizes the need to be aware of other mental health services available, and highlights on the need for the peer supporters to look after themselves. Chapter 3 speaks about providing basic psychological support for NTDs. This chapter promotes the value and practices of good communication and advises on how to maintain this with affected people to achieve the needed behavioral change and their acceptance to receive care. This chapter briefs the peer supporters on how to prepare, especially by improving their knowledge base, for helping the affected people. The important issue that there may be particular forms of attention needed for specific groups is also addressed; the component of this chapter guides how to support the specific groups like children, adolescents, elderly persons, persons with disabilities or at the risk of discrimination or violence. The chapter not only explains how to help, but also how the peer supporters should end support. This chapter provides details about three important action principles of BPS-N (identical to PFA)–“Look, Listen and Link” (Table 2).

**Table 2:** Core concepts and practices of the BPS-N Guide.

**LOOK**	✓ **Check for safety.** ✓ **Check for people with obvious urgent basic needs.** ✓ **Check for people with serious distress reactions**
**LISTEN**	✓ **Approach people who may need support** ✓ **Ask about people’s needs and concerns** ✓ **Listen to people and help them to feel calm**
**LINK**	✓ **Help people address basic needs and access services** ✓ **Help people cope with problems** ✓ **Give information** ✓ **Connect people with loved ones and social support**

Chapter 4 gives guidance for the providers of help, e.g., steps that one can take as a part of getting ready to help. This includes advice for the peer supporters on how to manage their own stress through healthy work and life habits, as well as guidance on the value of rest and reflection. A pocket guide follows the last chapter (Supplement S3).

The chapters in BPS-N are similar to those in the two PFAs. The first chapter of Ebola PFA has been replaced with chapter on NTDs. Considering the importance of COVID, a chapter on COVID was also included. The second chapter of Ebola PFA “Understanding PFA” has been replaced by “Understanding BPS”. The group felt the peer supporters and the frontline workers should be expected to only provide basic psychological support as they are not trained counsellors. And this will also be easier to explain to the authorities in the Indian and other contexts. The content of the different chapters was re-written keeping the NTDs in mind. However, it followed the Look, Listen and Link principle of the PFA.

## Discussion

It has been globally recognized that mental health is a key issue in NTD work, and several core agreements and frameworks have been adopted, with clear goals to reduce the burden of NTDs and mental health conditions [15]. The global mental health initiatives have been emphasizing the promotion of human rights in mental health care [16]. However, mental health and human rights services have not been systematically integrated into the public health system and they have rarely been assessed for effectiveness [17]; one of the contributing factors here is the limited research capacity for such intervention research [18].The development of the new BPS-N tool, based on the WHO PFA, reflects an important step in bringing mental health support—as a right like any other social welfare service of the governments—closer to those in need of this form of care at the community level. It potentially can help to reduce the significant gaps between demand and supply for mental health care by guiding appropriate community-based responses to the mental health challenges, needs and entitlements of persons affected by NTDs.

The decision to use the PFA as the base document of the BPS-N was an important step. Although evidence of the effectiveness of PFA in different situations needs adequate documentation [19], it has been used widely and its merits have been described by multiple authors, and in more visible recent medical crises than in NTDs [19–24]. However, persons affected by NTDs do not normally face the humanitarian crises or life-threatening situations that usually cause severe, acute mental distress. Rather they must cope with the chronic stresses typically associated with disability, stigma, social exclusion and poverty. The consensus team therefore was careful to make adaptations that change the focus from acute help to the psychosocial support of people affected by chronic stresses.

Despite its name, PFA covers both psychological and social support, and how to provide it [8]. It is essentially a tool that anyone can learn and implement regardless of their training, making it especially appropriate for those settings and communities challenged by limited access to mental health care [24]. Individuals affected by NTDs are commonly very neglected, isolated and lonely as it is usual to find only few who are similarly affected in their communities. Preliminary evidence already suggests that the use of PFA training can significantly enhance the providers’ knowledge of the appropriate skills, and thus is likely to lead to increased ability of supporters to assist those affected by NTDs and reduce the current gap in mental health care [21]. Similarly, the new guide on BPS-N, more specifically designed for the affected persons (peers), aims to build relevant knowledge, capacities, compassion and confidence among these groups to help those with NTDs, often rejected, and in community settings with limited mental health services [13]. When it comes to mental health care for people affected by NTDs, there are many constraints in terms of infrastructure, services, staff, and capacity, especially in rural areas. In any given scenario of limitations, lay persons and volunteers from within the community, such as peers who have similar experiences as those needing help, could provide much-needed initial support on the basis of BPS-N tool. They cannot only fill the deficiencies, but also complement available professional services. BPS-N, therefore, may be a cost-effective and suitable solution to challenging conditions that force deprivation, inequality and social exclusion.

It is important to emphasize that the higher-level professional health assistance may need to be provided where it is available, and that the BPS-N only provides basic support. BPS-N is not intended to be a substitute for professional advice required to provide treatment for specific, often serious, medical or psychological conditions. The advice of a physician, psychologist or psychiatrist will be needed when the advice offered in this guide is not sufficient or does not lead to improvement. It is necessary for peer supporters to know their limits in providing mental health advice and care, and to refer to specialists wherever possible. They need to remember and be reminded that they should provide basic psychological support only, and not attempt any counselling or such other professional services like psychotherapy; such attempts could lead to challenging situations. They should be under careful supervision themselves and know when to refer.

The guide is based on the principle that peer supporters are drivers of change, and by using the BPS-N, they can reduce the impact of NTDs. The local peer supporters have a unique advantage as they share a common understanding and experience of the issues and needs of their peers besides a sense of oneness, common culture and community. This helps to improve connections, conversations and in finding solutions.

While the BPS-N guide aims at the peer supporters as actors, it can also be used by others involved in supporting persons living with physical and/or psychosocial consequences of NTDs. The guide can be a resource to build the knowledge and skills of general health care staff to give basic psychological support to persons affected by NTDs, and others with mental health issues. In turn, these staff can train lay persons and peer volunteers who will be able to help the affected persons. in the communities where mental health services are not available [13]. Peers, other community volunteers and grassroots health workers are better placed to provide BPS-N by linking their clients with both government services and community support.

The three action principles—Look, Listen, & Link—constitute the core of the BPS-N. The “Look’ principle urges the supporters to look for the safety, urgent basic needs and distress reactions of the affected persons. The ‘Listen’ principle highlights the need for looking into the issues of the affected by listening to them, and empathetically understanding them from their perspectives, and listening to the affected and excluded with the purpose to provide a solution. Demonstration of active, appreciative and respectful listening by looking into the eyes of the clients can build hopes and restore trust. This is a critical need as the persons affected by NTDs and mental health issues often feel that no one listens to them, and that they are left behind by society including their own community and kith and kin. The principle of ‘Link’ emphasizes the need to help a person make different levels of connections to claim or fulfil their rights and access entitlements. The BPS-N guide not only promotes “Look, Listen, and Link”, but also principles such as safety, dignity, rights, empathy, compassion and equity. Continuous training, awareness, behaviour change, and practical action based on these principles can provide much needed services and help in building trust, and a sense of security.

Persons physically affected by NTDs are not the only group who experience mental health problems (e.g., depression and anxiety), their children and other family members can also suffer similar issues [15,25,26]. This can arise from the stresses of caring for their NTD affected family members and from the general disruption to building a positive family environment. It may also include the effects arising from the inability of an NTD affected person to work and support their family. BPS-N provides tips on how to support persons who may need special care; this includes children and other family members, women, older people, adolescents, people with severe mental issues, and those who have been discriminated, or victims of violence. The BPS-N guide development faced two limitations. While the English guide was translated into the mainstream language of Hindi, it could not be translated into the local dialect (Khota) which some peer supporters and clients in the intervention area were more comfortable with. Translation could not be done because of lack of written script of the dialect. However, the language issue was tackled by the peer supporters who knew both Hindi and Khota. Another limitation was the lack of adequate involvement of the affected persons for their inputs as there were no community consultations in the field. They could not be engaged, to the extent planned, because of the pandemic. Challenged by the COVID 19; physical meetings and field visits were limited by the lockdowns. As most of the meetings were conducted virtually, the affected persons, without internet connectivity and equipment, could not have the chance to contribute to the process; only a few people in Delhi could join. However, whatever inputs were made by a small number of affected people, made the guide suitable for the study participants as we tested it during implementation of the intervention. The BPS-N protocol has currently been developed for use with persons affected by leprosy or LF. However, it is fundamentally a generic procedure, albeit with specific disease information in the introductory chapter which can, for use with other NTDs or other conditions, can simply be replaced by the relevant wording as appropriate for any public health issues.

However, it must also be said that the implementation of new protocols to the NTD care efforts such as those for leprosy and LF, no matter how important and obvious they may appear, can be difficult as has been seen with basic lymphedema care in LF where there are still many patients not aware or continuing to use, the basic hygiene protocol recommended by WHO [25]. There can be other challenges like poverty, and lack of education, willingness and skills on the part of the peers and volunteers. Implementation of a protocol like this requires constant attention, and concerted effort, training, and commitment by programs and communities for raising awareness, improving communication, building capacities, compassion, empathy and ownership. While this new, consensus and inclusive protocol can be used in the field, the resulting experiences will likely lead to various nuances of its implementation and adjustments to the new guide.

The future consequences of applying the guide will likely improve mental wellbeing and participation and reduce stigma for the persons with NTDs. In NLR operational states, at least one million people affected by NTDs can benefit from the application of the BPS-N guide. With its relevance for larger public health issues, and increasing realisation of mental wellbeing, the BPS-N tool offers promising consequences as it can build a better society, equity and inclusion, and promote community volunteering and giving which is beneficial for both the support recipients and providers.

While the guide has shown promising results [13]; as a next step, NLR India has been working on the integration of the tool in its programs through the trainings of the field staff, peer supporters, other community volunteers and leaders. Field visits, follow-up and supportive supervision are the approaches in order to ensure that the BPS-N providers are well capacitated. NLR India has also trained the general health care staff of the government, and shared the protocol with several interested people, national and international civil society organisations who sought advice and support on applying the guide. They have appreciated the relevance of the guide after several dissemination meetings and presentations by NLR India. NLR India’s positive response has been contributing to the promotion of the protocol, and programs though evaluations need to be planned. It is important that NTD program managers in endemic countries are made aware of the existence of this important protocol, and that those running training programs for care in these countries, e.g., staff of leprosy hospitals, those instructing filarial lymphedema patients in the WHO essential lymphedema package of care [25], are also aware and begin to learn, be trained on, and use the BPS-N. As there is a relatively poor attention currently given to providing mental health care to those suffering from NTDs, in the present focus to those with leprosy and filarial lymphedema, the BPS-N provides an important opportunity to reverse this situation.

On the basis of the experience and lessons from the application of the guide; the following training, capacity and motivation building approaches are recommended which can significantly enhance the providers’ knowledge, communication and other BPS-N skills: a.) regular trainings of the BPS-N providers, b.) peer learning and sharing of good practices and successes of peer support, c.) on-the-job trainings, and supportive supervision, d.) incentivising the BPS-N service providers who create and capacitate other service providers, e.) supporting trained BPS-N providers to deliver trainings to new recruits, and f.) awarding the learners and leaders of the Look, Listen, Link practices. The trainees should be able to use BPS-N, reduce stigma, and improve mental well-being.

It is recommended to use participatory training methodologies that emphasize on interaction, and practical applications through group discussions, role play and problem-solving exercises. This inclusive learning environment empowers the participants, and the learners can contribute their knowledge, experience and perspectives, and learn actively.

There is a need for further studies including on a methodological evaluation of the new BPS-N guide for understanding the feasibility and applicability of field implementation by the peer support groups with minimal training inputs. It is important that this new BPS-N guide now be tested in the field both by individual NTD care-giving groups, such as LF programs implementing as part of a LF essential package of care, and through structured randomized controlled trials (RCT). An RCT has already been planned by NLR India for establishing the effectiveness of the peer support approach-based BPS-N to address mental wellbeing, stigma and social participation of persons affected by leprosy and lymphatic filariasis in Jharkhand, India. Experiences of using this new tool should be regularly reviewed to validate and further advance the use of this protocol.

## Conclusion

The new BPS-N guide is a useful protocol, and easy to apply. While it has shown promising results, its application both in programs and through research is necessary. Drawing lessons from testing is important for a.) improving the protocol, and b.) encouraging more mental health care activities in the field. Any appropriate adjustments should be made through a similar consensus procedure described in this paper. The guide has been developed with a method that is replicable. It involved a stepwise process of actively engaging the experts and affected persons, who had knowledge, skills, lived experience and insights, and were willing to contribute to this guide resulting in the protocol as was planned. The collaborative adaptation and development of the BPS-N guide may be followed as a process model by the people and organisation interested in similar consensus and inclusive protocols.
